# Long-Term Complications and Quality of Life After Urinary Diversion for Bladder Cancer: A Systematic Review and Meta-Analysis

**DOI:** 10.7759/cureus.84744

**Published:** 2025-05-24

**Authors:** Fadi Abusal, Abdullah Alawadi, Anna Akpala, Saja Obeidat, Nabeel Al-Omari, Mazen Alsharief, Amr Shalaby, Zeeshan Shaukat, Debashis Sarkar

**Affiliations:** 1 Urology, Queen Elizabeth Hospital Birmingham, Birmingham, GBR; 2 International Postgraduate Medical Training Scheme (IPGMTS), University Hospitals Birmingham, Birmingham, GBR; 3 Pediatrics, Yarmouk University, Irbid, JOR; 4 Clinical Sciences, Yarmouk University, Irbid, JOR; 5 General Surgery, Diana Princess of Wales Hospital, Grimsby, GBR

**Keywords:** bladder cancer, continent cutaneous urinary diversion, ileal conduit (ic), orthotopic neobladder (onb), radical cystectomy (rc)

## Abstract

Urinary diversion after radical cystectomy (RC) for bladder cancer significantly influences long-term outcomes and patient quality of life. This systematic review and meta-analysis examined complications occurring five years or more post-RC across the three main diversion types: ileal conduit (IC), orthotopic neobladder (ONB), and continent cutaneous urinary diversion (CCUD). A total of 16 studies with 8,493 patients were included. We compared morbidity, mortality, reoperation, and urinary tract infection, along with patient-reported quality of life (QoL). The meta-analysis revealed that IC was associated with reduced urinary stone formation and improved global health scores, while better urinary function and body image perception were associated with ONB. Data on CCUD were limited, preventing meta-analysis, but qualitative findings suggested distinct functional and QoL benefits compared to IC and ONB. This study highlights the importance of individualized surgical decision-making based on long-term outcomes and patient preferences; however, the limited data on CCUD highlights the need for further high-quality research to clarify its long-term safety, functional outcomes, and QoL implications.

## Introduction and background

Bladder cancer is among the most prevalent urinary tract cancers, with an estimated 573,000 new cases and 213,000 deaths worldwide in 2020 [1]. While non-muscle-invasive bladder cancer is often managed conservatively, radical cystectomy (RC) remains the gold-standard treatment for muscle-invasive and high-risk disease [2]. RC, however, necessitates urinary diversion, a type of reconstructive surgery with a significant impact on long-term patient outcomes. The three most prevalent types of urinary diversion, ileal conduit (IC), orthotopic neobladder (ONB), and continent cutaneous urinary diversion (CCUD), are all popular, yet each has unique physiological and quality-of-life (QoL) implications [3].

Perioperative and short-term complications of such urinary diversions have been well described in the literature. Long-term complications (≥5 years), however, are less well characterized and variably reported, and it is unclear how they impact morbidity, QoL, and survival. In studies evaluating postoperative quality of life following urinary diversion, patient-reported outcomes are commonly assessed using validated instruments. Frequently used tools include the European Organisation for Research and Treatment of Cancer Quality of Life Questionnaire (EORTC QLQ-C30), which measures cancer-specific domains, and the 36-Item Short Form Survey (SF-36), a generic instrument assessing overall health-related quality of life. Risks of renal function deterioration, metabolic disturbances, ureteroenteric strictures, and secondary malignancies in patients undergoing urinary diversion may occur years following surgery and have a significant impact on long-term health outcomes [4-6]. Aside from physiological complications, urinary diversion also carries a considerable psychosocial burden, affecting body image, sexual function, and overall well-being [7,8]. The extent to which different diversion types affect these parameters in the long term is unclear, and a synthesis of available evidence is necessary. Although many studies have assessed the QoL in patients undergoing urinary diversion, comparative studies synthesizing both long-term complications and patient-reported outcomes are few [9,10]. The lack of standardized methodologies and heterogeneity in outcome definitions also preclude a clear understanding of the long-term safety and efficacy of these surgical strategies.

To the best of our knowledge, no review paper has looked specifically at the long-term complications associated with IC, ONB, and CCUD. This systematic review and meta-analysis will look at the long-term complications (≥5 years) associated with different types of urinary diversion- IC, ONB, and CCUD after RC for bladder cancer.

Research question

What are the long-term complications (≥5 years) associated with different types of urinary diversion (ileal conduit, orthotopic neobladder, and continent cutaneous diversion) after radical cystectomy for bladder cancer, and how do these complications impact patient quality of life and survival?

## Review

Methods

The present systematic review and meta-analysis were done following the guidelines presented in the Preferred Reporting Items for Systematic Review and Meta-Analysis (PRISMA) 2020 statement [11].

Data Sources and Search Strategy

A systematic literature search was conducted for articles published from 2000 to March 2025. This was to ensure the relevance of surgical techniques and outcomes. The index databases used were PubMed, ScienceDirect, Cochrane Library, and Google Scholar. Topic keywords were used to generate search strings (Table 1). The identified studies were then subjected to a study selection process.

**Table 1:** Search strings The search string for ScienceDirect was shortened because the database only accepts search strings with a maximum of eight Boolean operators. The search in ScienceDirect was limited to the title, abstract, and keywords to limit the number of irrelevant studies during searching.

Databases	Search strings
PubMed	("Urinary Diversion"[MeSH] OR "Ileal Conduit" OR "Orthotopic Neobladder" OR "Continent Cutaneous Diversion") AND ("Cystectomy"[MeSH] OR "Radical Cystectomy")
Cochrane Library	("Urinary Diversion" OR "Ileal Conduit" OR "Orthotopic Neobladder" OR "Continent Cutaneous Diversion") AND ("Radical Cystectomy" OR "Bladder Cancer Surgery")
ScienceDirect	("urinary diversion" OR "ileal conduit" OR "orthotopic neobladder" OR "continent cutaneous diversion") AND ("radical cystectomy" OR "bladder cancer surgery") AND ("long-term complications")
Google Scholar	"Urinary diversion" OR "ileal conduit" OR "orthotopic neobladder" OR "continent cutaneous diversion" AND "radical cystectomy" OR "bladder cancer surgery" AND "long-term complications" OR "late complications" OR "≥5 years outcomes"

Secondary Search

In addition to the search conducted on the three databases, a direct search was conducted using Google Scholar. This search was performed to identify relevant grey literature and additional studies that may not have been indexed in the main databases. Given Google Scholar’s algorithmic instability and lack of search reproducibility, it was not used as a primary source for systematic screening. Instead, it served as a supplementary tool to capture potentially relevant reports or institutional publications. We acknowledge its limitations and only used it to enhance the comprehensiveness of our review.

Inclusion and Exclusion Criteria

Studies were eligible for inclusion if they met the following criteria: (i) the study population had to be ≥18 years who underwent RC with urinary diversion for bladder cancer, (ii) Eligible interventions included IC, ONB, or CCUD, (iii) Studies needed to report on long-term complications (≥5 years post-surgery), (iv) Randomized controlled trials (RCTs), prospective or retrospective cohort studies, and case-control studies with a minimum follow-up period of five years were considered, and (v) Studies had to be written in the English language.

Studies were excluded if they had a follow-up period of < 5 years. Additionally, case reports, editorials, and review articles without original data were not considered. Studies involving pediatric populations or those examining cystectomy for conditions other than bladder cancer were excluded. Research that did not differentiate between types of urinary diversion was also ineligible. Non-English studies and those with incomplete or unclear outcome data were excluded from the review.

Two independent reviewers screened the titles and abstracts for all identified studies. Full-text articles were retrieved for studies that appeared to meet the inclusion criteria. Disagreements were resolved by consensus or through consultation with a third reviewer.

Data Extraction and Quality Assessment

Each article in the review was summarized in a table for study characteristics. The extracted data included Author and Year, Study Design, Country, Sample Size (N), Follow-Up Duration, Age (Years), Sex (M/F), Comorbidities, and Intervention Details. There was a separate table where the extracted outcome data were summarized according to the different urinary diversion interventions used.

The quality of the included studies was assessed using the Joanna Briggs Institute (JBI) quality appraisal tool, and publication bias was estimated using funnel plots [12].

Statistical Analysis

The statistical analysis was done using the Review Manager Version 5.4 software. Continuous variables were reported as mean ± standard deviation (SD), and statistical significance was set at p<0.05. For the meta-analysis of dichotomous data, the Mantel-Haenszel model was applied, while the inverse variance model was used for continuous data. Odds ratios (OR) were used for dichotomous outcomes and standardized mean differences (SMD) for continuous outcomes. Meta-analyses were conducted employing a random-effects model to account for potential heterogeneity across studies. Heterogeneity was evaluated using the I² statistic, with thresholds of 25%, 50%, and 75% representing low, moderate, and high heterogeneity, respectively. Funnel plots were utilized to assess the presence of publication bias.

Results

Literature Search

**Figure 1:**
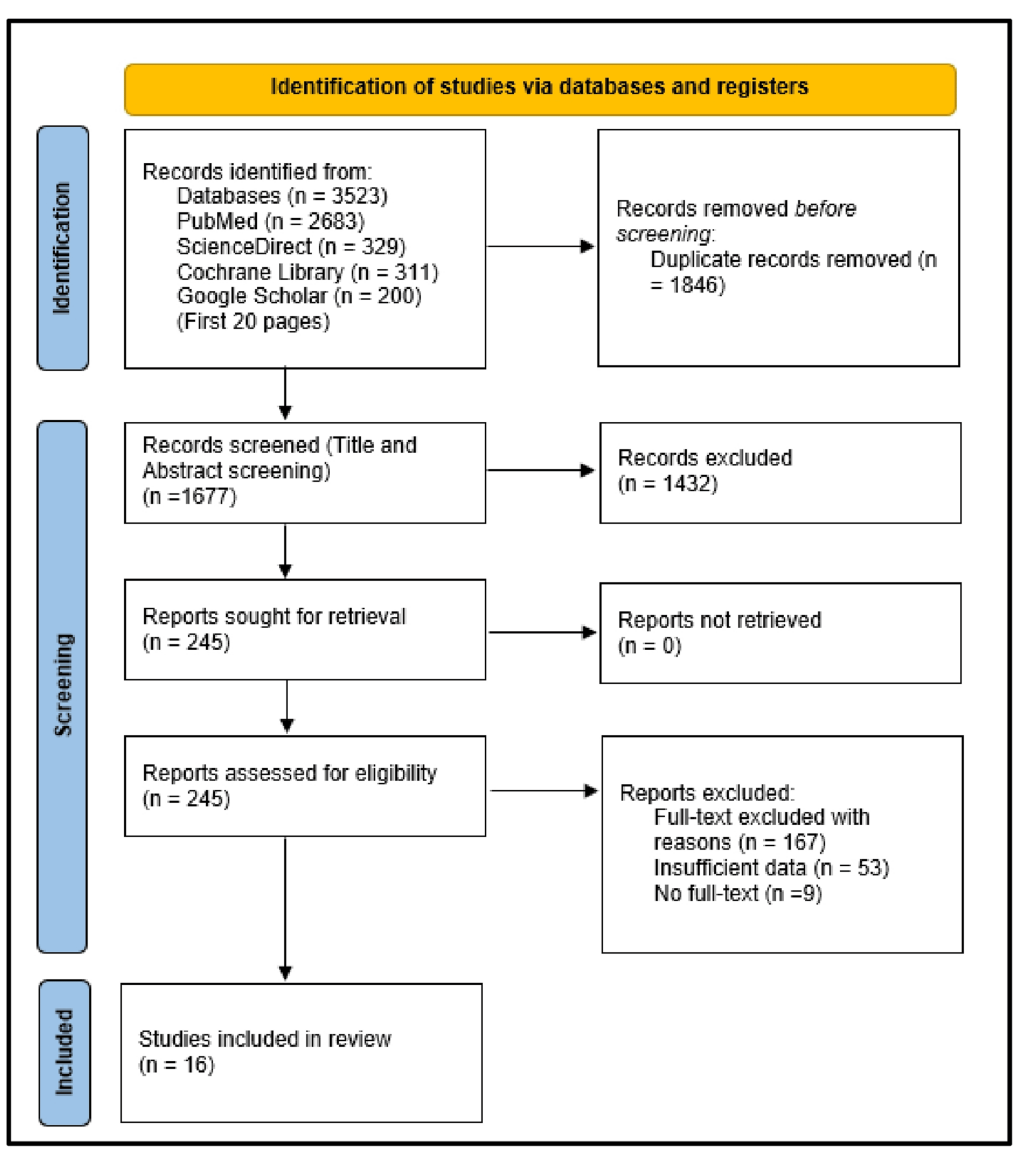
PRISMA flowchart showing the study selection process PRISMA: Preferred Reporting Items for Systematic Review and Meta-Analysis

In total, 3523 articles were identified, and 1846 duplicates were removed. During the article and abstract screening, 1432 were excluded following the eligibility criteria, and the remaining 245 articles were subjected to a full-text review. In total, 16 articles were included in the qualitative and quantitative analysis (Figure 1).

Quality Assessment

The quality of the included studies was evaluated using the JBI tool, a well-established and validated instrument for assessing research quality. We used the JBI Critical Appraisal Checklist for Case-Control Studies (2020 version) to assess methodological rigor. Among the case-control studies, 8 were rated as ‘fair,’ while another 8 received a ‘good’ overall quality rating (Table 2). In total, these studies demonstrated high methodological quality with a low risk of bias. This strengthens confidence in the reliability of our findings and supports the robustness of our research conclusions.

**Table 2:** JBI clinical appraisal results for case-control studies Item 1: Were the groups comparable other than the presence of disease in cases or the absence of disease in controls? Item 2: Were cases and controls matched appropriately? Item 3: Were the same criteria used for identification of cases and controls? Item 4: Was exposure measured in a standard, valid and reliable way? Item 5: Was exposure measured in the same way for cases and controls? Item 6: Were confounding factors identified? Item 7: Were strategies to deal with confounding factors stated? Item 8: Were outcomes assessed in a standard, valid and reliable way for cases and controls? Item 9: Was the exposure period of interest long enough to be meaningful? Item 10: Was appropriate statistical analysis used? Comments: Yes/No/Unclear/NA JBI: Joanna Briggs Institute

Study	Item 1	Item 2	Item 3	Item 4	Item 5	Item 6	Item 7	Item 8	Item 9	Item 10	Overall
Aboumarzouk et al., (2014) [13]	Yes	Yes	Yes	Yes	Yes	Yes	Yes	Yes	Yes	Yes	Good
Al Awamlh et al., (2015) [14]	Yes	Yes	Yes	Yes	Yes	Yes	Yes	Yes	Yes	Yes	Good
Chevarria et al., (2024) [15]	Yes	Yes	Yes	Yes	Yes	Yes	Yes	Yes	Yes	Yes	Good
Collins et al., (2013) [16]	Yes	Yes	Yes	Yes	Yes	No	No	Yes	Yes	Yes	Fair
El Khoury et al., (2024) [17]	Yes	Yes	Yes	Yes	Yes	Yes	Yes	Yes	Yes	Yes	Good
Erber et al., (2012) [18]	Yes	Yes	Yes	Yes	Yes	Yes	Yes	Yes	Yes	Yes	Good
Gellhaus et al., (2017) [19]	Yes	Yes	Yes	Unclear	Unclear	Yes	Yes	Yes	Yes	Yes	Fair
Gore et al., (2010) [20]	Yes	Yes	Unclear	Unclear	Unclear	Unclear	Unclear	Unclear	Unclear	Unclear	Fair
Hedgepeth et al., (2010) [21]	Yes	Yes	Yes	Yes	Unclear	Unclear	Unclear	Yes	Yes	Yes	Fair
Huang et al., (2015) [22]	Yes	Yes	Yes	Yes	Yes	Yes	Yes	Yes	Yes	Yes	Good
Kim et al., (2014) [23]	Yes	Yes	Yes	Yes	Yes	No	No	Yes	Yes	Yes	Fair
Pattou et al., (2022) [24]	Yes	Yes	Yes	Yes	Yes	Yes	Yes	Yes	Yes	Yes	Good
Sogni et al., (2008) [25]	Yes	Unclear	No	Unclear	Yes	No	No	Yes	Yes	Yes	Fair
Su et al., (2020) [26]	Yes	Yes	Yes	Yes	Yes	No	No	Yes	Yes	Yes	Fair
van-Hemelrijck et al., (2013) [27]	Yes	Yes	No	Yes	No	Yes	Yes	Yes	Yes	Yes	Fair
Wong et al., (2024) [28]	Yes	Yes	Yes	Yes	Yes	Yes	Yes	Yes	Yes	Yes	Good

Baseline Characteristics

The studies included in this review provided data on a total of 8,493 patients (n= 5853 for IC, n=1989 for ONB, and n= 651 for CCUD). IC was reported in 14 studies, ONB in 12 studies, and CCUD in four studies. A summary of study characteristics is presented in Table 3. The review included five prospective case-control studies and 11 retrospective case-control studies. Reported patient demographics included age, male-to-female ratio, and comorbidities. The mean patient age was 64.9 ± 8.8 years, with follow-up durations ranging from 5 to 10.4 years. The studies were conducted across various regions: Poland, the United States, the United Kingdom, Sweden, France, Germany, Korea, Italy, China, and one multicenter data from the Asian consortium.

**Table 3:** Summary of included studies in the review for IC, ONB, and CCUD RC: radical cystectomy; NR: not reported; TNM: tumor node mode; M: Male; F: female; N: sample size; IC: ileal conduits; ONB: orthotopic neobladder; CCUD: continent cutaneous urinary diversion; ^^: same as what has been written in the box above signifying the use of more than one type of urinary diversion

Author (s) and Year	Study Design	Country	Sample Size (N)	Follow-Up Duration	Age (Years) (Mean ± SD)	Sex (M/F)	Comorbidities	Intervention Details		
								Type of Urinary Diversion	Surgical Approach	Use of Bowel Segment
Aboumarzouk et al., 2014 [13]	Prospective case-control	Poland	39	60 months after RC	60 ± 7.11	34/5	NR	IC	Laparoscopic	Ileal
^^	^^	^^	24	60 months after RC	57 ± 8.68	24/0	NR	ONB	Laparoscopic	Ileal
Al Awamlh et al., 2015 [14]	Prospective case-control	USA	170	60 months after RC	76	141/29	Hypertension= 107 (62%) and Diabetes mellitus= 36 (21%)	IC	Open and Robotic	Colonic
^^	^^	^^	73	60 months after RC	67.5	49/24	Hypertension= 42 (57%) and Diabetes mellitus= 13 (17%)	CCUD	Open and Robotic	Colonic
Chevarria et al., 2024 [15]	Retrospective case-control	United Kingdom	264	120 months after RC	68.3	194/70	Hypertension= 44.2% and Diabetes mellitus= 14.7%	IC	Open and Robotic	Ileal
Collins et al., 2013 [16]	Prospective case-control	Sweden	43	60, 72, 84, 96 and 108 months	69.9 ± 6.7	31/11	NR	IC	Robotic-assisted	Colonic
^^	^^	^^	70	60, 72, 84, 96 and 108 months	59.8 ± 9.0	62/8	NR	ONB	Robotic-assisted	Colonic
El Khoury et al., 2024 [17]	Retrospective case-control	France	24	Median follow-up was 62.5 months	59	5/19	NR	CCUD	Laparoscopic robot-assisted	Ileal
Erber et al., 2012 [18]	Retrospective case-control	Germany	23	60 months after RC	70	98/48	NR	IC	Open	Ileal
^^	^^	^^	34	60 months after RC	62	110/5	NR	ONB	Open	Ileal
Gore et al., 2010 [20]	Retrospective case-control	USA	1252	60 months after RC	NR	NR	NR	IC	NR	NR
^^	^^	^^	109	60 months after RC	NR	NR	NR	ONB	NR	NR
Kim et al., 2014 [23]	Retrospective case-control	Korea	161	75, 100, and 125 months after RC	67.1 ± 8.9	115/32	Diabetes = 7 (10.6%) and Hypertension = 41 (25.5%)	IC	Open	Ileal
^^	^^	^^	147	75, 100, and 125 months after RC	59.4 ± 9.4	156/5	Diabetes = 21 (14.3%) and Hypertension = 49 (33.3%)	ONB	Open	Ileal
Pattou et al., 2022 [24]	Retrospective case-control	France	14	Median follow up was 5.4 years	56.9 ± 9	10/4	NR	CCUD	Open	Ileal and colonic
Sogni et al., 2008 [25]	Prospective case-control	Italy	53	60 and 70 months after RC	78.9	NR	NR	IC	NR	NR
^^	^^	^^	32	60 and 70 months after RC	77.5	NR	NR	ONB	NR	NR
Su et al., 2020 [26]	Retrospective case-control	China	409	60, 90, 120, 150, and 180 months	63	400/9	NR	IC	NR	NR
^^	^^	^^	409	60, 90, 120, 150, and 180 months	63	397/12	NR	ONB	NR	NR
Gellhaus et al., 2017 [19]	Retrospective case-control	USA	44	At least 60 months after RC	67.2 ± 9.4	35/9	NR	IC	NR	NR
^^	^^	^^	48	At least 60 months after RC	58.4 ± 9.1	47/1	NR	ONB	NR	NR
Hedgepeth et al., 2010 [21]	Prospective case-control	USA	85	72, 96 months after RC	71.09 ± 8.23	66/19	NR	IC	NR	NR
^^	^^	^^	139	72, 96 months after RC	60.76 ± 9.30	116/23	NR	ONB	NR	NR
Huang et al., 2015 [22]	Retrospective case-control	China	78	60 months after RC	64	16/62	Hypertension= 28 (35.9%) and Diabetes= 16 (20.5%)	IC	Laparoscopic	Ileal
^^	^^	^^	39	60 months after RC	63.6	7/32	Hypertension= 9 (23.1%) and Diabetes= 7 (17.9%)	ONB	Laparoscopic	Ileal
van Hemelrijck et al., 2013 [27]	Retrospective case-control	Sweden	2918	72, 96, and 120 months after RC	66.4 ± 8.4	NR	NR	IC	NR	NR
^^	^^	^^	731	72, 96, and 120 months after RC	60.6 ± 9.6	NR	NR	ONB	NR	NR
^^	^^	^^	540	72, 96, and 120 months after RC	60.7 ± 9.8	NR	NR	CCUD	NR	NR
Wong et al., 2024 [28]	Retrospective case-control	Asia	314	60, 80, and 100 months after RC	69.2 ± 9.0	262/52	Diabetes mellitus= 87 (26.1%)	IC	Laparoscopic robot-assisted	NR
^^	^^	^^	207	60, 80, and 100 months after RC	61.6 ± 9.2	193/14	Diabetes mellitus= 48 (23.8%)	ONB	Laparoscopic robot-assisted	NR

Complication Outcomes

The complication outcomes observed across the included studies covering renal function, infection rates, stone formation, functional and anatomical issues, oncological outcomes, QoL assessments, survival and mortality rates, and reoperation frequencies are reported in Table 4.

**Table 4:** Complication outcomes IC: ileal conduits; NB: neobladder; CC: continent cutaneous urinary diversion; py: per year; UTI: urinary tract infection; NR: not reported; OS: overall survival; CSS: cancer-specific survival; BCI: bladder cancer index; BIS: body image scale; QoL: quality of life; ^^: same as what has been written in the box above signifying the use of more than one type of urinary diversion

Study	Urinary Diversion	Outcome Data							
		Renal Function, n (%)	Infections, n (%)	Stone Formation	Functional and Anatomical Issues, n (%)	Oncological Outcomes, n (%)	QoL scale	Survival and Mortality	Reoperation Rates, n (%)
Aboumarzouk et al., (2014) [13]	IC	NR	Sepsis =2 (5)	NR	Urine retention =0 (0); Urine leak =0 (0); Ureteral stenosis =8 (20.5); lymph leak =2 (5)	NR	NR	NR	12(39)
^^	ONB	NR	Sepsis =1 (4)	NR	Urine retention =1 (4); Urine leak =3 (12.5); ureteral stenosis =4 (16.7); lymph leak=1 (4)	NR	NR	NR	1(24)
Al Awamlh et al., (2015) [14]	IC	Decline in renal function = 58 (34)	NR	NR	NR	NR	NR	NR	NR
^^	CCUD	Decline in renal function = 24 (33)	NR	NR	NR	NR	NR	NR	NR
Chevarria et al., (2024) [15]	IC	NR	Pyelonephritis (6.4%), recurrent UTI (19.1%)	NR	Ureteral stricture (7.9%)	NR	NR	Mortality rates were 43.2% and 54.1% at five years and 10 years, respectively.	NR
Collins et al., (2013) [16]	IC	NR	NR	NR	NR	7(16.3)	NR	OS was 66.6% and CSS was 67.3%	NR
^^	ONB	NR	NR	NR	NR	13(18.6)	NR	OS was 66.6% and CSS was 67.3%	NR
El Khoury et al., (2024) [17]	CCUD	NR	NR	NR	Uretero-ileal stenosis= 3 (12.5)	Recurrence at 5 years= 1(4)	BCI	NR	62%
Erber et al., (2012) [18]	IC	NR	NR	NR	NR	NR	EORTC QLQ-C30 and BLM30	OS was 46% and 30% at five years and 10 years, respectively.	NR
^^	ONB	NR	NR	NR	NR	NR	EORTC QLQ-C30 and BLM30	OS was 67% and 46% at five years and 10 years, respectively.	NR
Gore et al., (2010) [20]	IC	NR	11 (2)	Urinary stones = 21 (4)	Stomal= 35 (6), Hernia= 58 (11), and Fistula formation= 20 (4)	NR	NR	NR	NR
^^	ONB	NR	2 (4)	Urinary stones = 7 (13)	Stomal= 5 (9), Hernia= 8 (14), and Fistula formation= 6 (11)	NR	NR	NR	NR
Kim et al., (2014) [23]	IC	NR	NR	NR	NR	NR	NR	OS was 38% at five years	NR
^^	ONB	NR	NR	NR	NR	NR	NR	OS was 59% at five years	NR
Pattou et al., (2022) [24]	CCUD	NR	NR	NR	Continence 11 (79), Cutaneous tube stenosis 1(7)	NR	BCI	NR	NR
Sogni et al., (2008) [25]	IC	NR	NR	NR	NR	NR	EORTC QLQ-C30 and BLM 30	NR	NR
^^	ONB	NR	NR	NR	Continence 4 (25), urine retention 2 (12.5)	NR	EORTC QLQ-C30 and BLM 30	NR	NR
Su et al., (2020) [26]	IC	NR	NR	NR	NR	NR	NR	OS and CSS were 55% and 72.9% at five years, respectively	NR
^^	ONB	NR	NR	NR	NR	NR	NR	OS and CSS were 45.1% and 66.0% at five years, respectively	NR
Gellhaus et al., (2017) [19]	IC	NR	NR	NR	NR	NR	BCI	NR	NR
^^	ONB	NR	NR	NR	NR	NR	BCI	NR	NR
Hedgepeth et al., (2010) [21]	IC	NR	NR	NR	NR	NR	BCI and EORTC BIS	NR	NR
^^	ONB	NR	NR	NR	NR	NR	BCI and EORTC BIS	NR	NR
Huang et al., (2015) [22]	IC	NR	NR	NR	NR	NR	BCI and BIS	NR	NR
	ONB	NR	NR	NR	NR	NR	BCI and BIS	NR	NR
van Hemelrijck et al., (2013) [27]	IC	Hydronephrosis incidence per 1000-py =38.74	UTI incidence per 1000 py = 130.61	Incidence per 1000-py = 19.74	NR	NR	NR	Death; Incidence per 1000-py = 181.62	NR
^^	ONB	Hydronephrosis incidence per 1000-py = 43.49	UTI incidence per 1000 py = 139.62	Incidence per 1000-py = 37.46	NR	NR	NR	Death; Incidence per 1000-py = 84.88	NR
^^	CCUD	Hydronephrosis incidence per 1000-py = 52.17	UTI incidence per 1000 py = 131.61	Incidence per 1000-py = 52.51	NR	NR	NR	Death; Incidence per 1000-py = 98.19	NR
Wong et al., (2024) [28]	IC	NR	NR	NR	NR	NR	NR	Disease-free survival was 55% at five years. Overall survival was 60% at five years	NR
^^	ONB	NR	NR	NR	NR	NR	NR	Disease-free survival was 70% at five years. Overall survival was 70% at five years	NR

Long-term complication outcomes

Morbidity and Mortality

**Figure 2:**
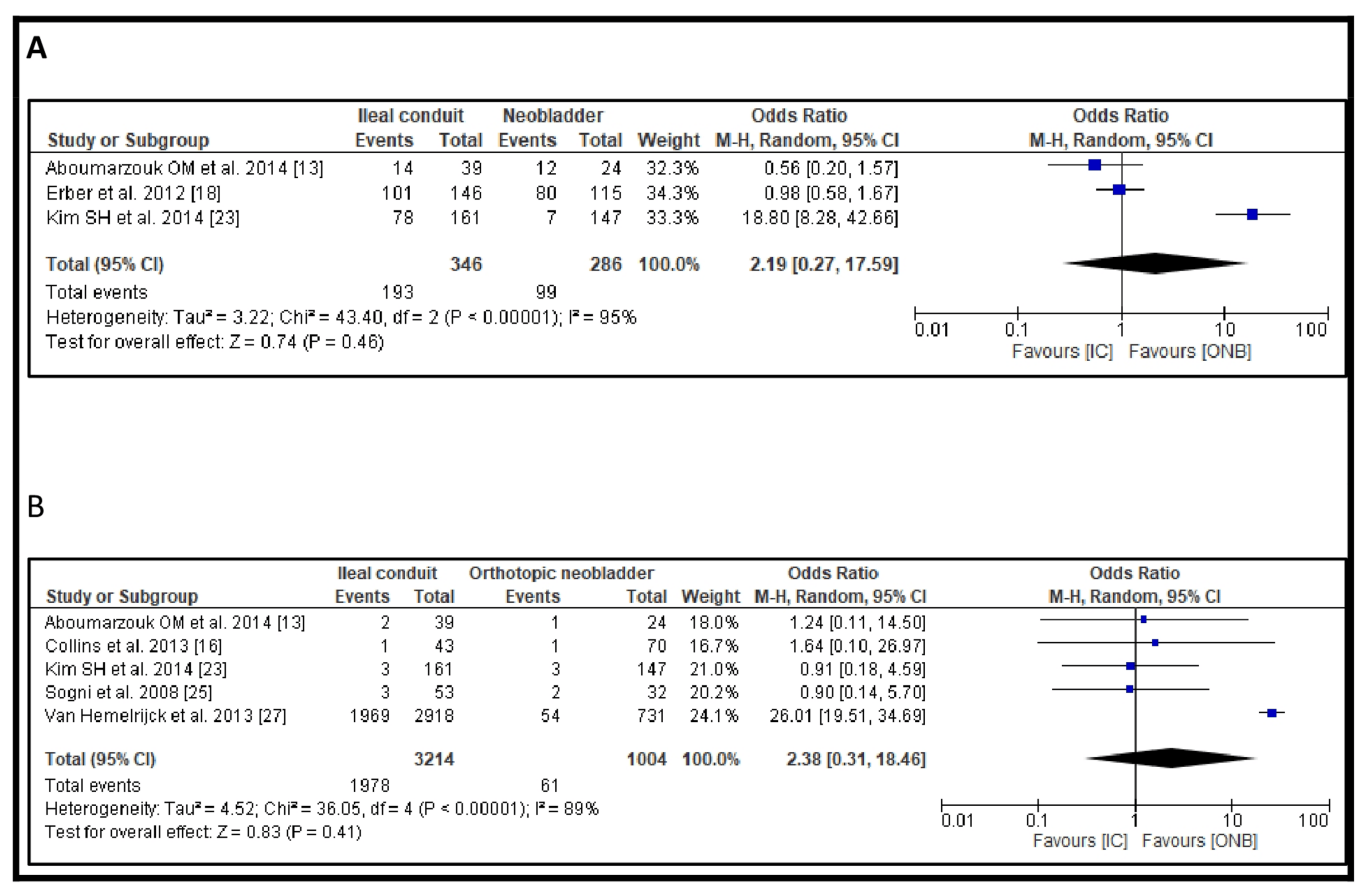
Long-term complications: (A) Incidence of postoperative morbidity and (B) postoperative mortality

**Figure 3:**
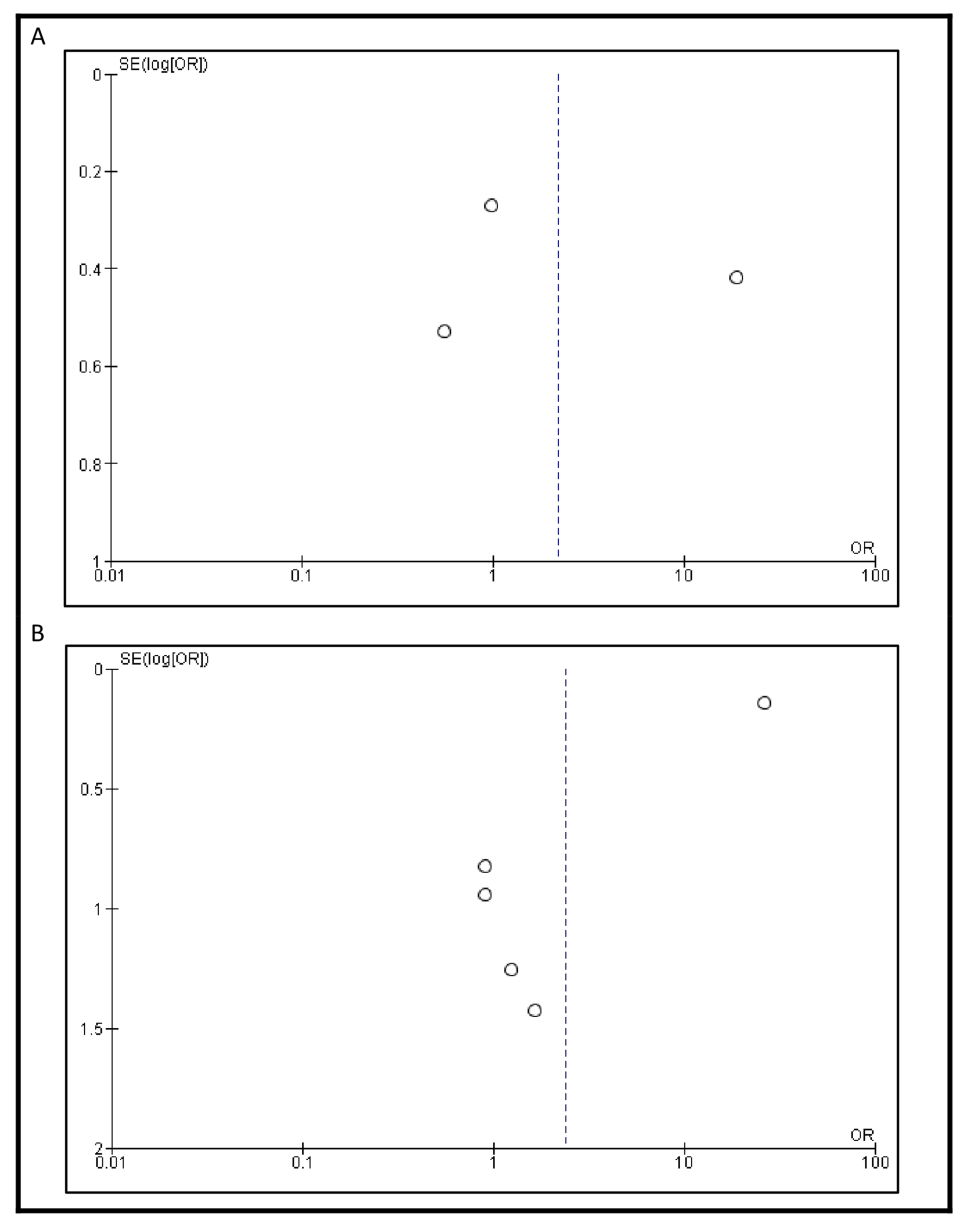
Funnel plots showing publication bias for (A) incidence of postoperative morbidity and (B) postoperative mortality Each dot represents a study included in the respective meta-analysis. A displays the funnel plot for morbidity outcomes, based on studies [13], [18], and [23], while B shows the funnel plot for mortality outcomes, based on studies [13], [16], [23], [25], and [27]. The vertical lines represent the pooled effect estimates, and the diagonal lines indicate the 95% confidence limits

Postoperative morbidity was reported in three studies. The incidence was higher in patients who underwent IC urinary diversion compared to those who received an ONB (193/346, 55.8% vs. 99/286, 34.6%). However, the difference was not statistically significant (OR 2.19, 95% CI: 0.27-17.59, p=0.46) (Figure 2A). Postoperative mortality was reported in four studies. The mortality rate was greater in patients with IC urinary diversion than in those who underwent ONB (1978/3214, 61.5% vs. 61/1004, 6.1%). However, this difference was also not statistically significant (OR 2.38, 95% CI: 0.31-18.46, p=0.41) (Figure 2B). Funnel plots showing publication bias for morbidity and mortality are shown in Figure 3.

Measures of Long-Term Durability

**Figure 4:**
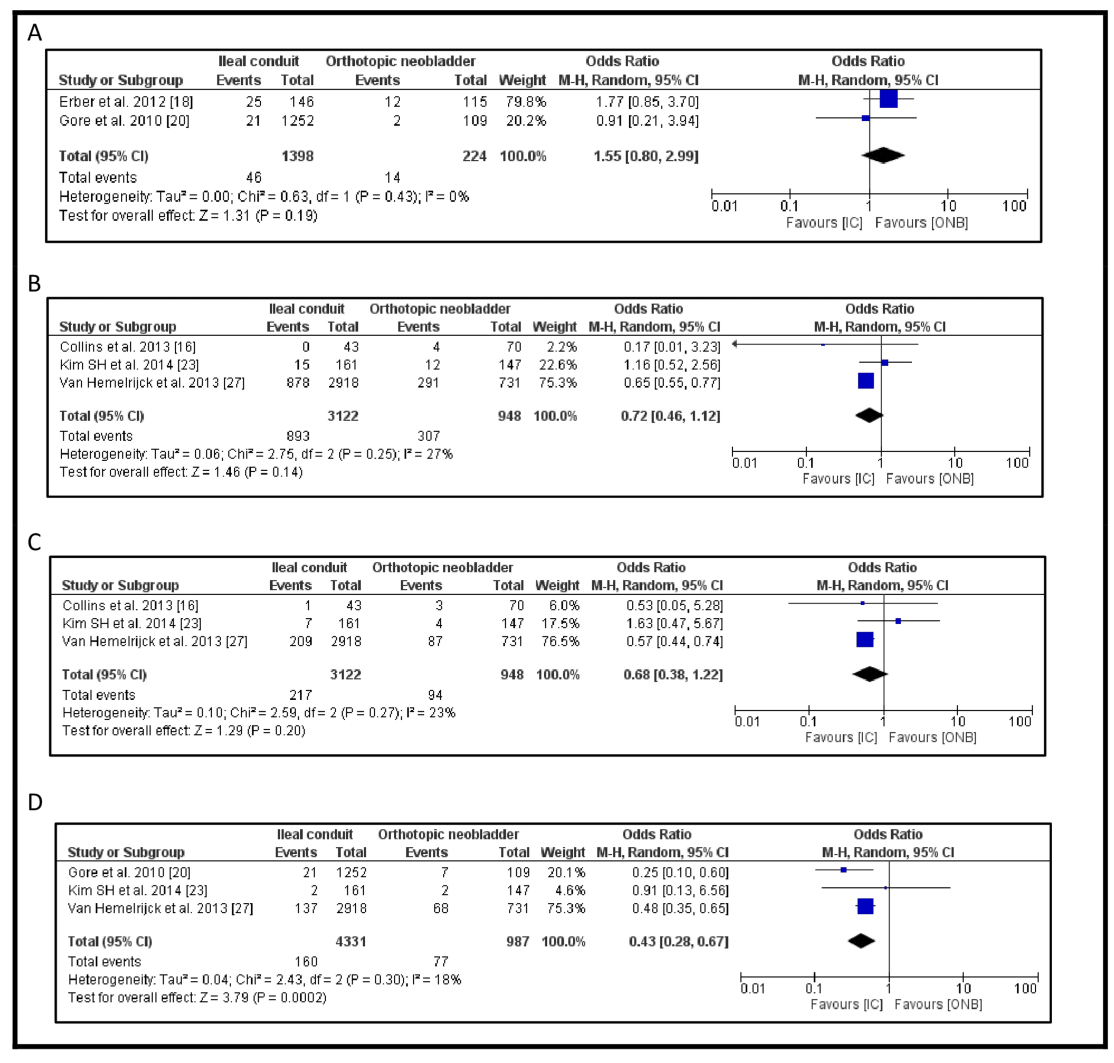
Secondary outcome in the IC vs ONB: (A) rate of re-operation, (B) incidence of UTI, (C) incidence of ureteric stricture and (D) incidence of urinary stones IC: ileal conduit; ONB: orthotopic neobladder; UTI: urinary tract infection

The long-term durability outcomes assessed in this review included re-operation rates, urinary tract infection (UTI) rates, urinary stone formation, and ureteric stricture rates. The meta-analysis results for these outcomes are detailed in Figure 4. Among the 16 studies included, two examined re-operation rates. The re-operation rate was higher in patients with an IC compared to those undergoing ONB formation (OR 1.55, 95% CI: 0.80-2.99, p=0.19); however, this difference was not statistically significant (Figure 4A).

UTI incidence was reported in three studies. Patients with IC had a lower incidence of UTIs compared to those with ONB (893/3122, 28.6% vs. 307/948, 32.4%), but the difference was not statistically significant (OR 0.72, 95% CI: 0.46-1.12, p=0.14) (Figure 4B).

Ureteric stricture rates were also reported in three studies. The incidence was lower in patients undergoing IC compared to ONB (217/3122, 7.0% vs. 94/948, 9.9%), but the difference did not reach statistical significance (OR 0.68, 95% CI: 0.38-1.22, p=0.20) (Figure 4C).

**Figure 5:**
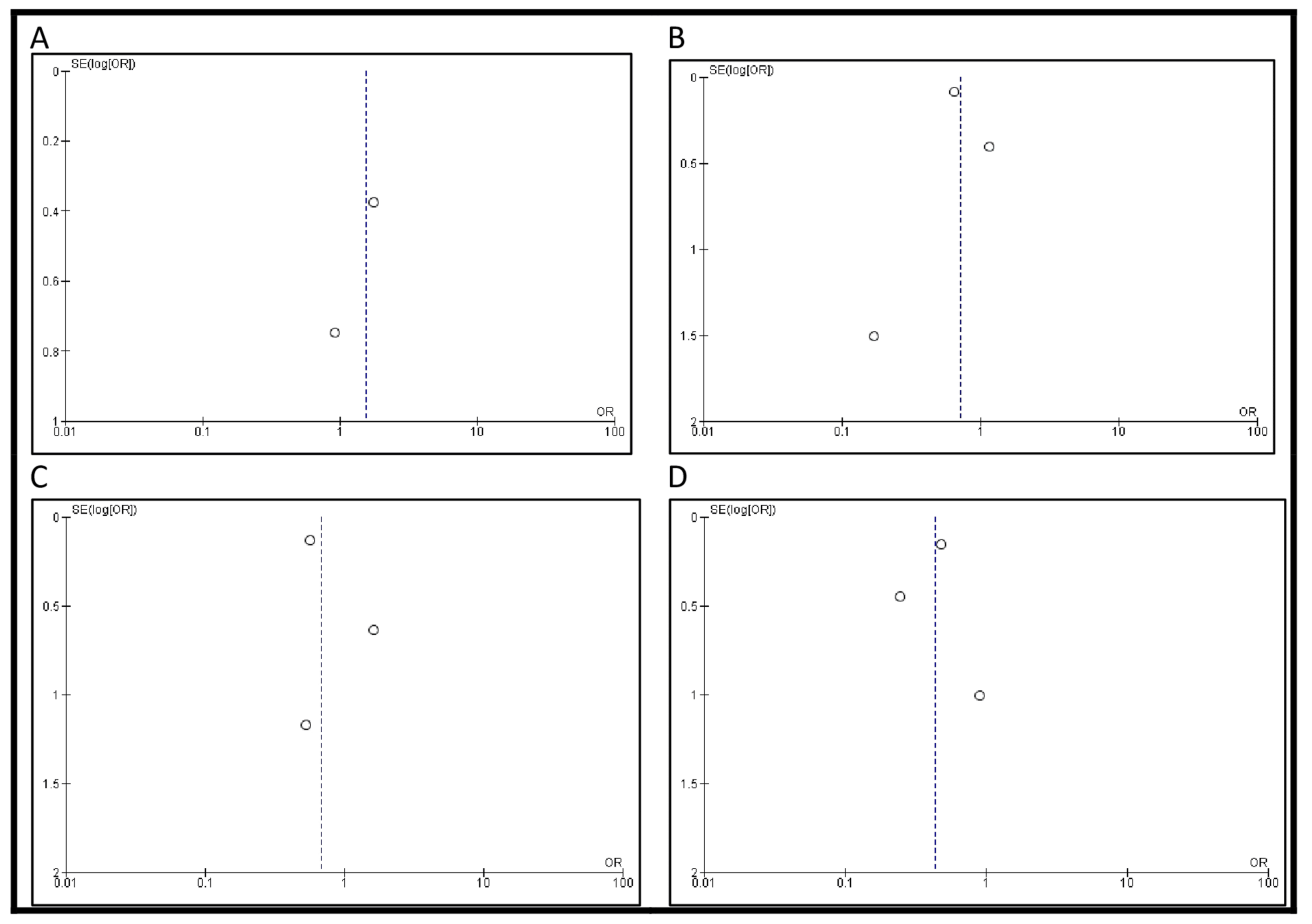
Funnel plots showing publication bias for (A) rate of re-operation, (B) incidence of UTI, (C) incidence of ureteric stricture, and (D) incidence of urinary stones A-D display funnel plots for the rate of re-operation, incidence of urinary tract infection (UTI), ureteric stricture, and urinary stones, respectively. All plots are based on studies [16], [18], [20], [23], and [27]. Vertical lines indicate pooled effect estimates; diagonal lines represent 95% confidence limits

In contrast, urinary stone formation was significantly lower in patients with IC compared to those with ONB (160/4331, 3.7% vs. 77/987, 7.8%). This difference was statistically significant (OR 0.43, 95% CI: 0.28-0.67, p=0.0002) (Figure 4D). Publication bias of the four outcomes is shown in Figure 5.

Quality of Life

**Figure 6:**
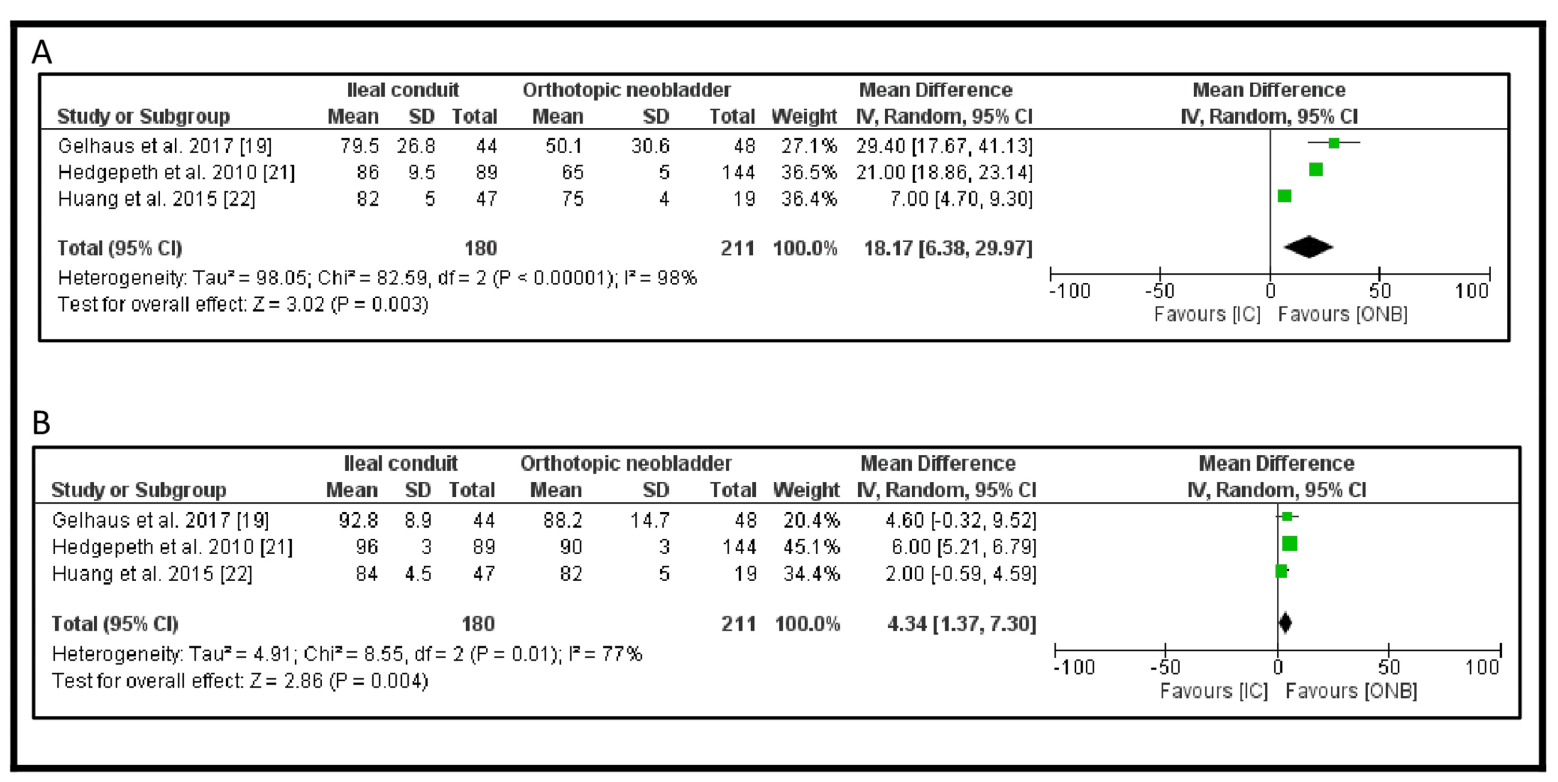
BCI subdomain scores the IC vs. ONB cohorts: (A) urinary function subdomain score and (B) urinary bother subdomain score IC: ileal conduit; ONB: orthotopic neobladder; BCI: bladder cancer index

**Figure 7:**
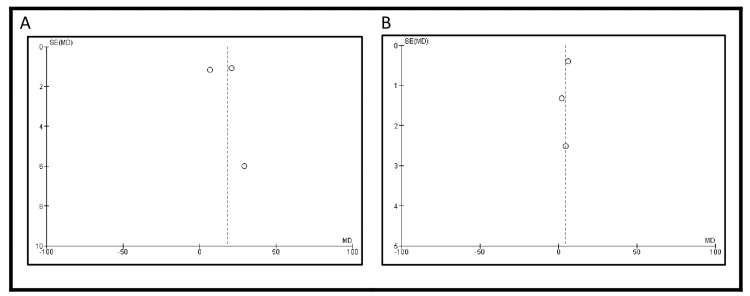
Funnel plots showing publication bias (A) urinary function subdomain score and (B) urinary bother subdomain score A shows the funnel plot for urinary function scores; B shows that for urinary bother scores. Both are based on studies [19], [21], and [22]. Vertical lines indicate pooled effect estimates; diagonal lines represent 95% confidence limits

The bladder cancer index (BCI) score for urinary function was assessed in three studies [19,21,22], including a total of 391 patients (Figure 6A). The pooled analysis using a random-effects model showed a significant improvement in urinary function for patients with an ONB compared to those with an IC, with a mean difference of 18.17 (95% CI: 6.38-29.97, p=0.003). However, there was considerable heterogeneity among the studies (I² = 98%) (Figure 7A).

Similarly, the BCI score for urinary bother was reported in three studies [19,21,22], also including 391 patients (Figure 6B). The pooled results indicated a significant advantage for ONB, with a mean difference of 4.34 (95% CI: 1.37-7.30, p=0.004). However, substantial heterogeneity was present (I² = 77%) (Figure 7B).

**Figure 8:**
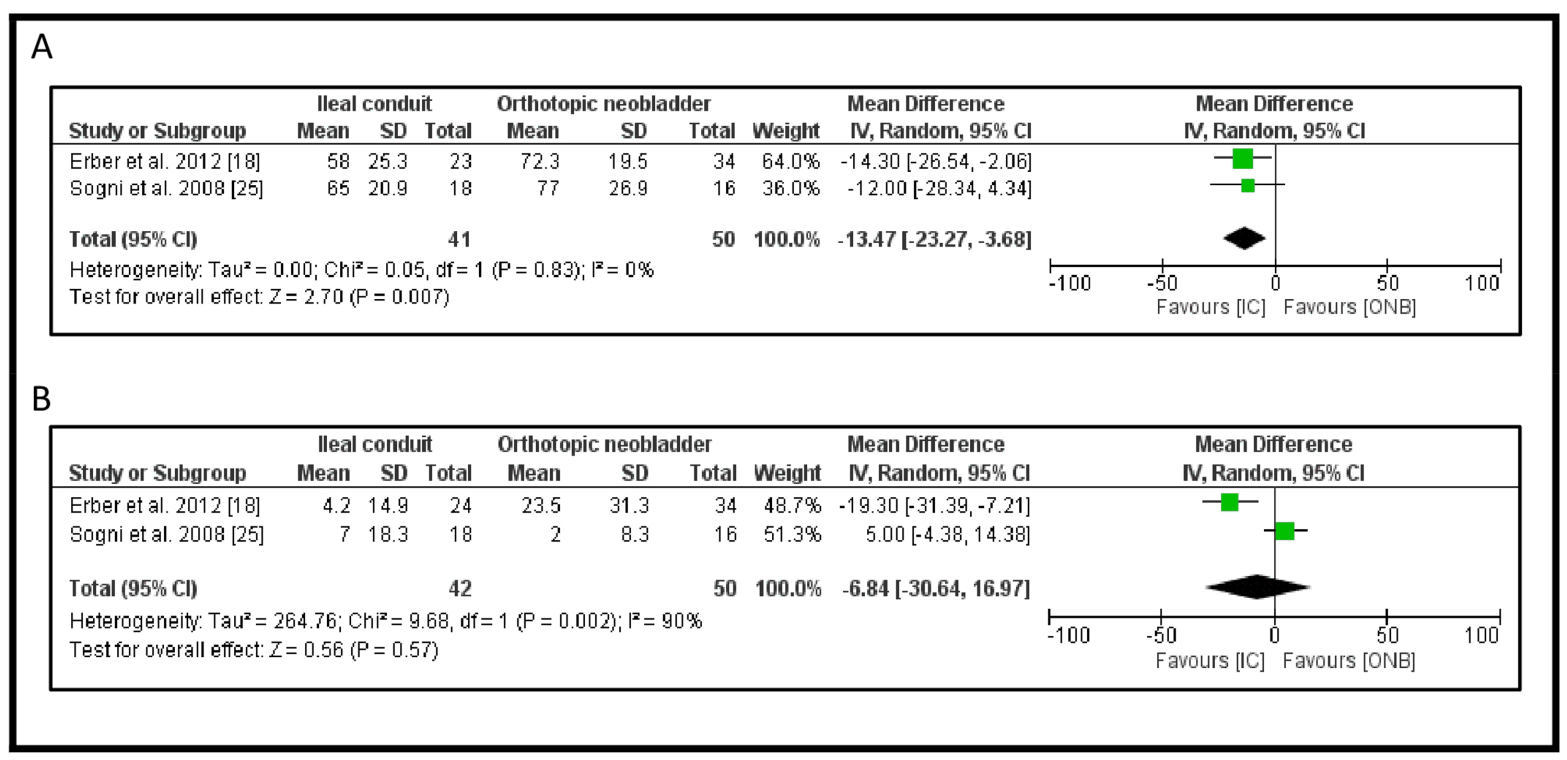
EORTC QLQ-C30 subdomain scores: (A) global health status/quality of life and (B) diarrhea symptoms EORTC QLQ-C30: European Organisation for Research and Treatment of Cancer Quality of Life Questionnaire

**Figure 9:**
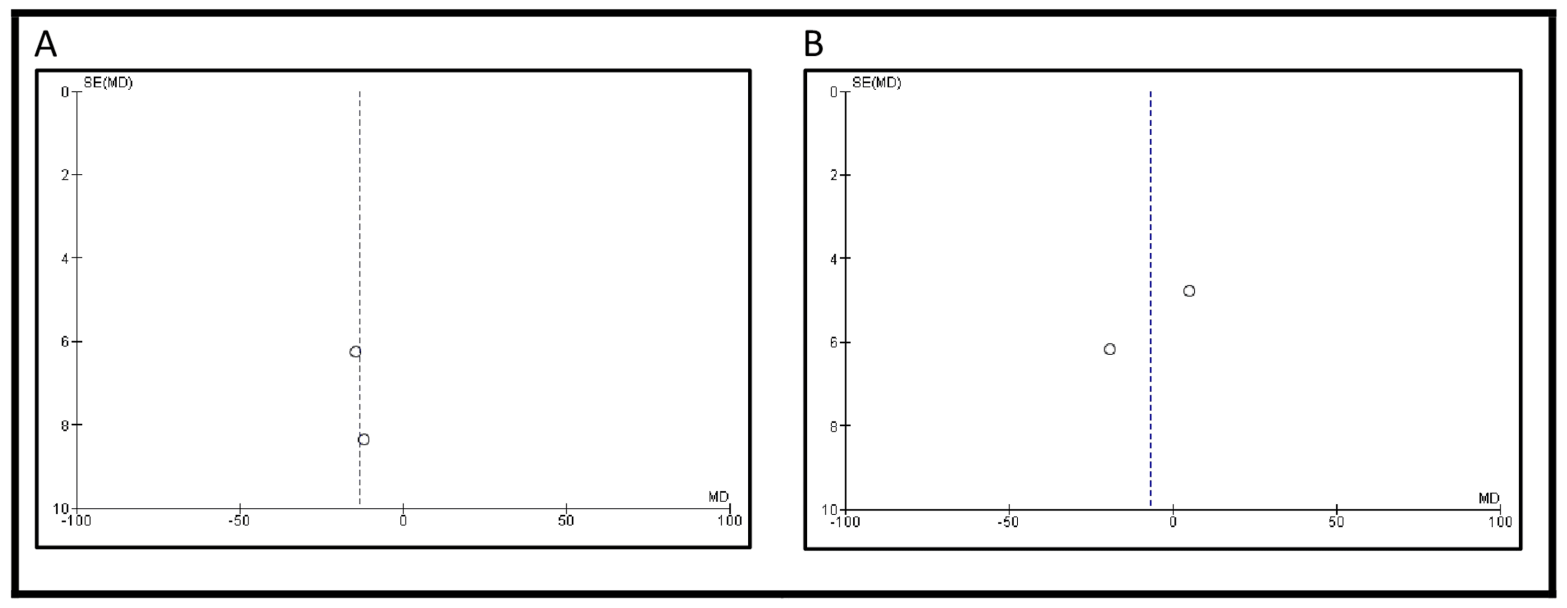
Publication bias: (A) funnel plot for global health status/quality of life and (B) funnel plot for diarrhea subdomain scores A displays the funnel plot for global health status/quality of life; B for diarrhea symptoms. Both are based on studies [18] and [25]. Vertical lines indicate pooled effect estimates; diagonal lines represent 95% confidence limits

The EORTC QLQ-C30 score for global health status/quality of life was assessed in two studies [18,25], including a total of 91 patients (Figure 8A). The pooled analysis using a random-effects model showed a significant difference of -13.47 in favor of the IC over the ONB (95% CI: -23.27 to -3.68, p=0.007). This result is statistically significant (I² = 0%) (Figure 9A).

The QLQ-C30 score for diarrhea symptoms was reported in two studies [18,25], including 92 patients (Figure 8B). The pooled results demonstrated a difference of -6.84 in favor of IC, indicating fewer diarrhea-related symptoms compared to ONB (95% CI: -30.64 to -16.97). However, this result was not statistically significant (p=0.57). There was considerable heterogeneity among the studies (I² = 90%) (Figure 9B).

**Figure 10:**
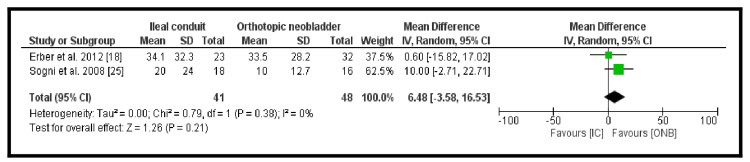
Forest plot for the body image QLQ-BLM30 subdomain score

**Figure 11:**
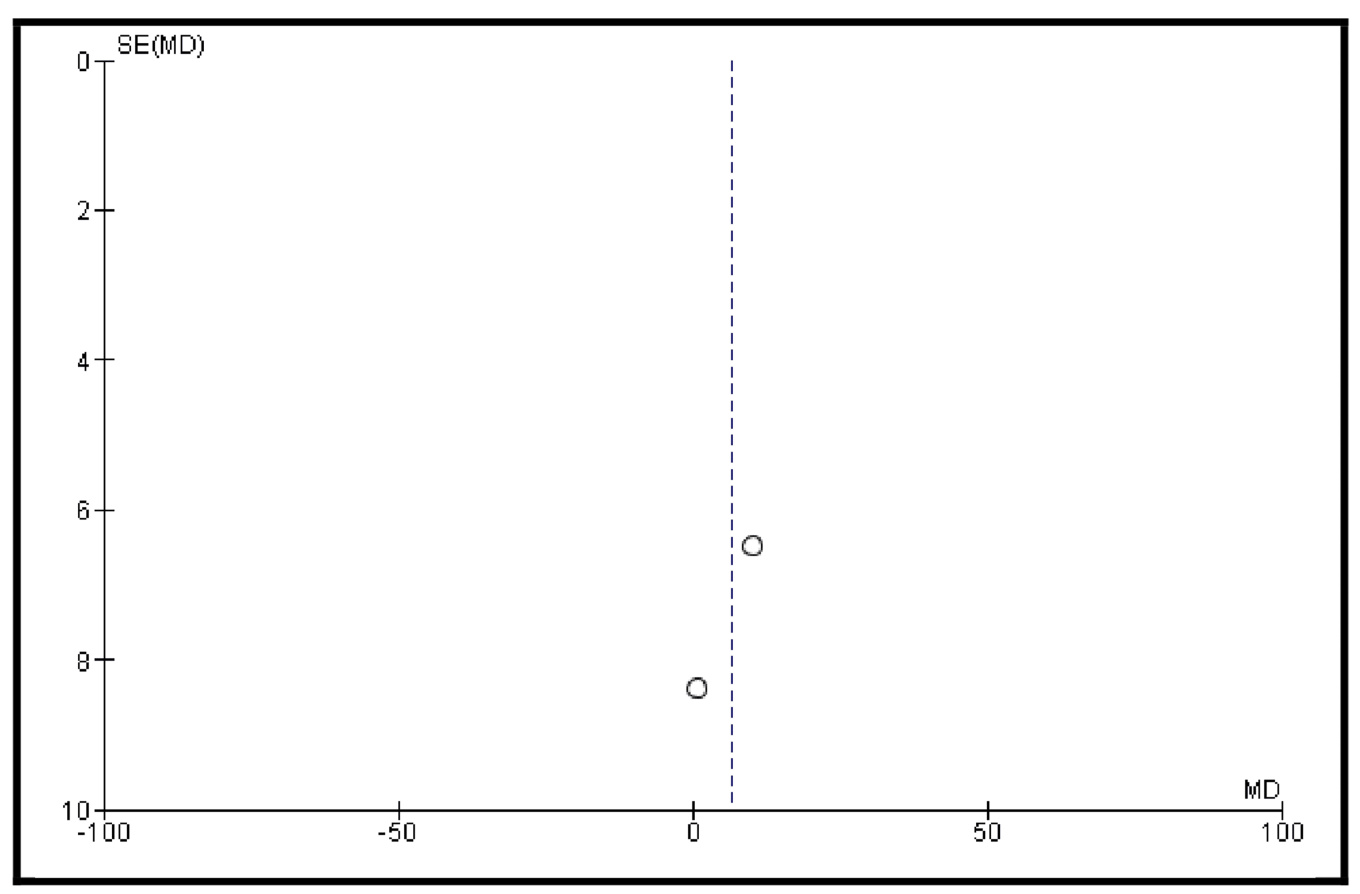
Publication bias for the body image QLQ-BLM30 subdomain score The figure shows the body image QLQ-BLM30 subdomain score, based on studies [18] and [25]. The vertical line indicates the pooled effect estimate; diagonal lines represent 95% confidence limits

The QLQ-BLM30 score for body image was assessed in two studies (Figure 10). The pooled analysis showed a difference of 6.48 in favor of the ONB over the IC (95% CI: -3.58 to 16.53). However, the results are not statistically significant (I² = 0%) (Figure 11).

Long-Term Qualitative Outcomes for CCUD

Reports on CCUD showed both its advantages and risks. van Hemelrijck et al. showed that the risk for UTIs was greater in patients with a continent cutaneous reservoir compared to those with an ileal conduit (HR: 1.11, 95% CI 0.94-1.30) [27]. CCUD and ONB were also associated with greater risks of wound and abdominal wall hernias, urinary tract stones, hydronephrosis, nephrostomy tube treatment, and renal failure. Al Awamlh et al. also showed that while patients who had ileal conduit had poorer preoperative renal function, postoperative renal outcome and diversion-related complications were comparable for IC and CCUD [14].

Functionally, Pattou et al. directly compared two CCUD techniques, ileal pouch with Mitrofanoff/Monti’s principle vs. Miami ileocolic pouch, and detected no difference in continence rate, capacity for self-catheterization, late complications, or patient satisfaction overall [24]. El Khoury et al. (2024) suggested that CCUD could be a suitable alternative to ileal conduit (Bricker diversion) for patients with urethral invasion or at increased risk of neobladder incontinence [17]. These findings demonstrate that while CCUD can be offered to carefully selected patients, its risks and complications need to be weighed carefully against its functional and QoL advantages.

Discussion

Summary of Findings

This systematic review and meta-analysis compared long-term (≥5 years) complications of IC, ONB, and CCUD following RC. Meta-analysis revealed that ONB had more favorable BCI urinary function and bother scores than IC, whereas IC had fewer urinary stones. There were no differences in reoperation rates, UTI incidence, or ureteric stricture rates between IC and ONB. A qualitative synthesis of CCUD findings showed comparable renal function to IC but increased risk of UTIs and abdominal wall complications. Our findings also demonstrated significant differences between these urinary diversions in long-term morbidity, renal function, and health-related quality of life (HRQoL).

Patients who receive RC are at risk for progressive renal function loss and increased mortality, with age and preoperative estimated glomerular filtration rate being the main risk factors [15]. Our results showed that IC was associated with a significantly reduced risk of postoperative complications compared with ONB and CCUD, with a reduced risk of UTIs, nephrostomy tube management, and renal failure. In addition, patients with IC had better urinary function scores compared with ONB [19,21], although urinary bother was similar in both groups. When QoL results are taken into consideration, the pooled analysis of EORTC QLQ-C30 scores showed that global health status was significantly better in IC patients compared with ONB. Similarly, IC was associated with significantly lower rates of symptoms of diarrhea. ONB, however, had a significant advantage regarding the perception of body image, implicating that while functional outcomes may favor IC, the psychological and social benefits of ONB cannot be ignored. Other authors have reported that ONB may be acceptable in elderly patients without resulting in additional morbidity compared with IC, with both diversions reporting acceptable QoL scores in the long term [25].

Our study also included qualitative findings for CCUD, which offers an alternative in cases where ONB is not feasible. CCUD was associated with an increased risk of UTIs, wound complications, and metabolic complications in the form of hydronephrosis and nephrostomy tube placement [27]. Some studies, however, reported comparable renal function outcomes between CCUD and IC, and no differences in continence rates or late complication rates between different CCUD configurations [14,24]. These findings suggest that CCUD remains an alternative, although additional research is needed to clarify its long-term impact. Survival outcomes were more dependent on patient factors than on the diversion type itself. While diversion type was found to influence late complications, it did not significantly alter overall morbidity rates when comparing IC and ONB [23]. Younger, male patients with lower tumor stages (T2) were also more likely to undergo ONB and were more likely to have a survival chance [26].

Clinical Implications

Given the lower risk of complications and improved urinary function outcomes, IC remains a viable choice for many patients, particularly those with high comorbidities or fears of postoperative complications. The psychological benefits of ONB, however, particularly its positive impact on body image and social reintegration, suggest that it needs to be offered as a choice for appropriately selected patients, particularly younger patients with superb preoperative renal function and without contraindications. CCUD is a great alternative when ONB is not feasible, particularly in patients with urethral involvement. Its increased risk of urinary and metabolic complications, however, requires prudent patient selection and long-term follow-up to reduce these risks. Research efforts should be aimed at optimizing surgical technique and postoperative management strategies to enhance the outcomes of CCUD. Finally, our findings underscore the need for patient-centred urinary diversion choice. Age, preoperative renal function, oncologic prognosis, and patient preferences are considerations to be taken into account when counselling patients on their surgical options. Shared decision-making, both on functional and psychosocial grounds, will be essential in optimizing patient outcomes and QoL after RC.

Study Limitations

One of the primary strengths of this review is the inclusion of long-term follow-up data (≥5 years), allowing for a robust assessment of complications and QoL outcomes in the long term. Since many postoperative complications, such as worsening kidney function, UTIs, and metabolic disturbances, manifest over time, the long-term follow-up of the studies included makes our findings more valid. Additionally, the synthesis of quantitative and qualitative evidence contributes to the clinical relevance of this review, providing an overall picture of urinary diversions beyond perioperative outcomes and short-term morbidity. All these strengths increase the generalizability of our findings, providing a balanced perspective for both clinicians and patients in the selection of a urinary diversion type.

However, this review is not without its limitations. Among the most significant challenges was heterogeneity among studies, including differences in patient selection criteria [27], and reporting of complications. Such heterogeneity limits the comparability of results and can contribute to bias in pooled estimates. Some studies also included small sample sizes [13,17,24], which can affect the accuracy of our meta-analysis results. Another significant limitation was the scarcity of CCUD data, which prevented quantitative synthesis for this diversion type. We were therefore compelled to rely on qualitative synthesis, which, while informative, lacks the statistical power of meta-analysis. The absence of large comparative studies on CCUD underscores the need for further research to better delineate its long-term risks and benefits. Despite these limitations, our review establishes a critical foundation for future research directed at optimizing urinary diversion selection and long-term patient outcomes.

Future Research Directions

Additional studies are necessary to fill gaps in the current evidence regarding urinary diversions, and in particular, CCUD outcomes. While our review indicates the potential of CCUD as an IC and ONB alternative, the lack of long-term data prevents firm conclusions. Large-scale multicenter cohort studies with long-term follow-up should be a priority in future studies to better establish the risks, benefits, and patient-reported outcomes of CCUD. Prospective comparisons of CCUD versus IC and ONB in heterogeneous patient groups, especially those with a high risk of neobladder incontinence or IC-related complications, are also necessary to better establish patient selection criteria.

RCTs are particularly needed to supply robust comparative evidence for CCUD because most studies are retrospective. RCTs would have the ability to reveal the influence of urinary diversion type on long-term renal function, metabolic complications, and HRQoL by reducing confounding factors that limit present analyses. Furthermore, standardization of complication reporting, functional outcomes, and patient satisfaction reporting across studies would render future meta-analyses more dependable, allowing more accurate comparisons between urinary diversion techniques. Having uniform outcome measures would also render the development of clinical guidelines more straightforward, which would ultimately improve decision-making for patients undergoing RC.

## Conclusions

This review provides a systematic evaluation of IC, ONB, and CCUD long-term complications following RC. Our findings demonstrated that IC offered a lower likelihood of urinary stone formation and long-term morbidity. CCUD appears to be a viable alternative in specific cases, particularly when ONB is not suitable; however, its long-term outcomes remain less established due to limited available data. ONB generally provides better functional outcomes, particularly in urinary function and bother, but is associated with a higher risk of complications compared to IC. These results highlight the need for individualized decision-making, not only based on age, renal function, and quality of life expectations, but also incorporating the physical and emotional limitations of each patient. It remains the responsibility of the experienced urological surgeon to guide this process, offering tailored recommendations that balance evidence-based outcomes with patient-specific needs and capabilities. Further research, including well-designed RCTs, is necessary to strengthen comparative evidence, particularly for CCUD, and to standardize reporting of long-term complications and functional outcomes.
